# Patient experience (PX) among individuals with disabilities in Japan: a mixed-methods study

**DOI:** 10.1186/s12875-022-01800-0

**Published:** 2022-07-26

**Authors:** Miho Iwakuma, Takuya Aoki, Mariko Morishita

**Affiliations:** 1 Department of Medical Communication, Graduate School of Medicine, Kyoto University, Yoshida-Konoe-cho, Sakyo, Kyoto, 606-8501 Japan; 2 Division of Clinical Epidemiology, Jikei University School of Medicine, 3-25-8 Nishishimbashi, Minato-ku, Tokyo, 105-8461 Japan; 3 Medical Education Center, Faculty of Medicine, Kyoto University, Yoshida-Konoe-cho, Sakyo, Kyoto, 606-8501 Japan

**Keywords:** JPCAT, PX, People with disabilities, Japan, mixed methods research (MMR)

## Abstract

**Background:**

People with disabilities (PWDs) tend to be disadvantaged in terms of receiving preventive medicine and medical checkups. About 7.6% of the Japanese population is estimated to have a disability. Although patient experience (PX) is an effective measure of patient-centeredness, little is known about the PX of PWDs. The present study aimed to compare the PX of PWDs with those of the non-disabled both quantitatively and qualitatively.

**Methods:**

The present study involved a questionnaire survey and a free-response question on the survey form. The quantitative part of the study involved a comparison of JPCAT scores between PWDs and non-disabled participants. JPCAT is composed of five primary care principles: First contact, Longitudinality, Coordination, Comprehensiveness (service provided and service available), and Community orientation. Descriptive statistics were used to assess age, sex, years of education, self-rated health status, and type of disability (for PWDs). Multivariable analysis was performed using a linear regression model to detect differences between PWDs and non-disabled participants in total and domain-specific JPCAT scores. The model included the following confounding variables: age, sex, years of education, and self-rated health status. The qualitative part of the study involved a thematic analysis of answers to the free-response question.

**Results:**

Data from 338 participants (169 PWDs and 169 non-disabled participants) were analyzed (response rate of 36% for PWDs). After adjusting for age, sex, years of education, and self-rated health status, PWD scores were significantly lower than those of non-disabled participants for the Longitudinality, Community Orientation, and Comprehensiveness (services available) domains of the JPCAT. Qualitative analysis yielded six themes, each of which was further divided to have Disability-Specific and General themes.

**Conclusions:**

JPCAT scores in PWDs were significantly lower than those of non-disabled participants for the Longitudinality, Community Orientation, and Comprehensiveness (services available) domains. Qualitative analysis revealed that PWDs shared several themes with non-disabled participants, but also to face unique challenges due to disabilities, such as the lack of a health care provider familiar with disabilities and the insurance transition at age 65, a unique feature of the Japanese health care system.

**Trial registration:**

The study was a non-interventional, observational research trial, and thus registration was not required.

## Background

### People with disabilities in health care studies

Compared to their non-disabled counterparts, people with disabilities (PWDs) in general are at a disadvantage in areas such as annual income, education level, work opportunities, housing, and mobility. In health care as well, the World Health Organization (WHO) has suggested that the health condition of PWDs is generally poorer than that of the non-disabled. This may reflect the unequal access to health care services and unmet needs of health care services, including promotion, prevention, and treatment [1].

Comparative research [2, 3] has found that, despite a drastic increase in life expectancy for PWDs, the sex, residential area (urban or rural), severity of the disability, and when individuals acquired disability (early/congenital or middle/late-onset) influence PWD life expectancies. For example, PWDs in China who live in urban areas or those with less severe disabilities had higher life expectancies than the non-disabled population, those with congenital physical disabilities had a much lower life expectancy, and men with congenital disabilities had a greater difference in life expectancy as compared to non-disabled men (17.1 years) than that observed in women with disabilities (12.7 years). A synthesis of literature study [4] targeting 36 selected studies conducted in low and middle-income countries revealed that PWDs in rural regions face four interrelated obstacles: acceptability, affordability, availability, and geography. Acceptability refers to attitudes of health care providers and the quality of care perceived by PWDs, and affordability relates to the ability of PWDs to pay for health care (both direct and indirect costs). Availability includes a subtheme of resources that is three-faceted: human resources, health services, and health care infrastructure. Finally, geography includes concepts such as the distance and transportation to a facility, as well as terrain and climate. These barriers do not independently exist, but converge. Therefore, the life expectancy or care quality of PWDs appears to be influenced by sex, severity of impairment, and regional differences in care.

In Japan, roughly 7.6% of the population is estimated to have some type of disability [5]. The number of people with physical, intellectual, and mental disabilities in Japan is 4.36 million, 1.09 million, and 4.19 million, respectively. Although some may have multiple disabilities, 34 per 1000 population have physical disabilities, 9 have intellectual disabilities, and 33 have mental disabilities. Japan uses a disability recordbook system, in which a recordbook is issued when a PWD applies for one. There are three types of recordbooks: physical disability recordbook, special education recordbook for children with disabilities, and health benefits recordbook for the mentally ill. Each recordbook has a classification called “disability grade” according to the degree of disability and symptoms in daily life. Although obtaining a disability recordbook is not mandatory, having one allows PWDs to receive various welfare services from Health and Welfare Services for Persons with Disabilities (HWSPWD).

Japan has two different insurance systems to support PWDs: HWSPWD and Long-term Care Insurance (LTCI). PWDs under the age of 65 receive services from the former. There are significant differences between the two systems. For example, mobility support, independence training, employment transition support, and employment continuation support are covered by HWSPWD. However, this coverage is lost at the age of 65, when HWSPWD is replaced by LTCI.

### Patient experience

Patient experience (PX) is the most effective measure of patient-centeredness, which emphasizes patient preferences, values, and needs, and is widely used as a quality indicator of primary care [6]. PX is used for the assessment of quality of care and is assessed by asking patients about events and perceptions in receiving care. Several PX instruments have been developed. One such instrument is the Primary Care Assessment Tool (PCAT), which is composed of five primary care principles: First contact, Longitudinality, Coordination, Comprehensiveness (service provided and service available), and Community orientation.

Previous studies have found that PX influences health outcomes through patient behavior [7, 8]. In Japan, PX has been used to study their associations with primary care practice location, specifically focusing on differences between hospital-based practices and community-based office practices [9], and to assess associations between social isolation and PX in elderly primary care patients [10]. However, PX of PWDs is largely unexplored, despite the fact that > 7% of the Japanese population is estimated to have a disability.

Against this backdrop, the present study aimed to compare PXs between PWDs and non-disabled people using scores from the Japanese version of PCAT (JPCAT), and to explore PX by qualitatively analyzing answers to a free-response question.

## Methods

### Design

The present study involved a questionnaire survey (JPCAT), and a free-response question included in the survey form. Among the three basic mixed methods research (MMR) designs (convergent, explanatory sequential, and explorative sequential) [11, 12], we used the convergent parallel design in which quantitative and qualitative data are analyzed separately and then integrated to provide additional information. Additionally, methodological triangulation was met by utilizing both qualitative and quantitative research.

### Procedures and settings

The questionnaire survey was distributed at several sites (an annual convention for people with spinal cord injuries, and a seminar hosted by the Center for Independent Living), and also through a mailing list from the Center for Independent Living to recruit PWDs. At these sites, the first author (MI) introduced herself and explained the research purpose to recruit participants. An e-mail survey was also sent to PWDs who had difficulties with writing. Upon returning the survey and when requested together with an inclusion of a written mailing address, a participant received a \500 incentive token. A total of 380 surveys were distributed through the aforementioned channels; 137 responses were received, giving a response rate of 36%. To enhance the response rate, a reminder was sent through a mailing list managed by the Center for Independent Living, resulting in 34 additional responses. Recruitment took place between 2018 and 2019.

Inclusion criteria for PWDs were the presence of a physical disability, age over 20 years, and the presence of a usual source of care. Adult PWDs, as well as non-disabled participants identified as having a usual source of care, were eligible for participation. To identify the usual source of care, we asked three questions: (1) Is there a doctor whom you usually go to if you are sick or need advice about your health?; (2) Is there a doctor who knows you best as a person?; and (3) Is there a doctor who is most responsible for your health care?. A respondent was considered to have a usual source of care if he or she answered yes to any of the three questions.

To assess PX of PWDs, we compared JPCAT scores of PWDs with those of non-disabled people, who were randomly selected from the Primary Care Organizations Reciprocal Evaluation Survey Study (PROGRESS) database [9] by one of the authors. PROGRESS was a cross-sectional survey conducted in 2018 that involved 25 Japanese facilities (six small- and medium-sized hospitals and 19 community clinics in both urban and rural areas) at which self-administered questionnaires were distributed to all outpatients aged ≥ 20 years who visited a primary care department in one of the participating facilities within a week of the survey period. Patients who were seen for the first time at the participating facilities were excluded because it was difficult for them to assess PX. Patients with severe mental disorders, such as advanced dementia, were also excluded [13].

### Quantitative analysis

The questionnaire included a demographic section, validated outcome measure for quantitative analysis, as well as ample free text space at the end to enable participants to comment on their experiences based on the following free-response prompt: “If you have any comments or concerns regarding aging or daily life in general, please feel free to write them in the space provided below.” The demographic section requested information on age, sex, education level, and type of disability, as well as the severity of disability as recorded on disability recordbooks.

The outcome measure used in the study was the JPCAT, which has been shown to be reliable and valid in previous studies [6]. The primary outcome measure was the JPCAT total score, which assesses five primary care principles: First contact, Longitudinality, Coordination, Comprehensiveness (service provided and service available), and Community orientation. These principles are described in more detail below.

First contact: Care services must be accessible and used by the population whenever a new need or problem arises. The JPCAT mainly assesses PX regarding out-of-hours care in primary care settings. Longitudinality: Longitudinality refers to the longitudinal use of a regular source of care over time, regardless of the presence or absence of disease or injury. The JPCAT mainly assesses whether patients feel that their primary care physician recognizes them as a whole person. Coordination: Coordination refers to the availability of information regarding all prior and existing problems and services, and the recognition of that information. The JPCAT mainly assesses PX in the context of previous specialist referrals. Comprehensiveness (services available): This principle refers to the availability of a wide range of services in primary care and their appropriate provision across a wide spectrum of care needs. Under “services available,” the JPCAT mainly assesses whether patients feel that they can receive care for mental health, dementia, and advanced care planning if necessary. Comprehensiveness (services provided): Under “services provided,” the JPCAT mainly assesses PX regarding appropriate advice about daily habits in the past. Community orientation: Community orientation refers to care that is delivered in the context of the community. The JPCAT mainly assesses PX regarding home visits and whether the patient feels that their primary care physician is interested not only in their individual health problems but also problems in the community [14].

The score for each domain is computed as the mean value for all converted scale scores in that domain. As a result, JPCAT scores range from 0 to 100 points, with higher scores indicating better performance. The total score is calculated from the mean of the four domain and two sub-domain scores and provides an overall measure of the quality of primary care principles. Both total scores and domain/subdomain scores are used as continuous measures for quantitative analysis. Additionally, a 3-point increase in PX, measured on a linear scale from 0 to 100, has been associated with a reduction in disenrollment from health plans, and also with advance care planning discussions with primary care professionals. Thus, a difference exceeding 3 points is considered a clinically meaningful difference [9].

### Statistical analysis

Descriptive statistics were used to analyze participant characteristics by age, sex, years of education, and self-rated health status. Multivariable analysis was performed using a linear regression model to detect differences in JPCAT domain/subdomain and total scores between PWDs and non-disabled participants. The model included the following potential confounders: age, sex, years of education, and self-rated health status. All covariates were evaluated as categorical variables.

For each analysis, we used a two-sided significance level of *P* = 0.05. Missing data on independent and dependent variables were addressed by applying multiple imputations (20 imputations) by fully conditional specification. Statistical analyses were performed using R version 3.6.3 (R Foundation for Statistical Computing, Vienna, Austria; www.R-project.org).

### Qualitative data and analysis

For the qualitative part of the study, contents of free-response answers were analyzed by thematic analysis. Investigator triangulation [15] of the qualitative data analysis was performed by a researcher with a communication studies background (MI) and a researcher with a family medicine background (MM). MI first deductively analyzed the free-comment text data using the five JPCAT domains. Next, MM independently read the same text data several times and reviewed the thematic categorizations made by MI. MM inductively suggested the inclusion of the primary care principle of “Accountability.” Finally, MI and MM discussed the validity of all qualitative themes while referring to the quantitative results, and came to a consensus. This investigator triangulation enabled us to double-check and mutually validate the qualitative results through a process of “cumulative validation” [16].

## Results

### Quantitative analysis

Data from a total of 338 participants (169 PWDs and 169 non-disabled participants) who were confirmed to have a usual source of care were analyzed. Disabilities of the 169 PWDs included cerebral palsy, cerebrovascular accident, cerebral contusion, spinal cord injury, spinal cord disease, amputation, neuromuscular disease, and bone dysplasia. The PWDs’ on-site response rate to the survey was 36%. In comparisons with non-disabled participants, we excluded two PWDs who were aged < 20 years. Table 1 summarizes the demographic data of participants.

**Table 1:** Participant characteristics (*N* = 338)

Characteristic (number, %)	Total *N* = 338 (%)		PWDs *N* = 169 (%)		Non-disabled *N* = 169 (%)	
Sex						
Male	165	43	112	66.3	53	31.4
Female	117	30.5	54	32	63	37.3
Missing data	56	26.6	3	1.8	53	31.4
Age (years)						
21–49	25	6.5	24	14.2	1	0.6
30–39	29	7.6	26	15.4	3	1.8
40–49	39	10.2	31	18.3	8	4.7
50–59	46	12	34	20.1	12	7.1
60–69	74	31.3	40	23.7	34	20.1
≥ 70	75	7.6	14	8.3	61	36.1
Missing data	50	25	0	0	50	29.6
Education						
Less than high school	52	13.5	19	11.2	33	19.5
High school	117	30.5	79	46.7	38	22.5
Junior college	43	11.2	21	12.4	22	13
College or more	61	15.9	38	22.5	23	13.6
Missing data	65	28.9	12	7.1	53	31.4
Self-rated health						
Very good	14	3.6	12	7.1	2	1.2
Good	66	17.2	39	23.1	27	16
Neutral	151	39.1	76	45	75	44.4
Poor	46	12	33	19.5	13	7.7
Very poor	9	2.3	7	4.1	2	1.2
Missing data	52	25.8	2	1.2	50	29.6

**Table 2:** Associations between disability and JPCAT scores (*N* = 338)

Outcome^a^	Unadjusted	*P*-value	Adjusted^b^	*P*-value
	mean difference (95% CI)		mean difference (95% CI)	
JPCAT				
Total score	− 7.01 (− 9.95 to − 4.07)	< 0.001	− 4.53 (− 8.39 to − 0.68)	0.022
First contact	− 4.63 (− 10.04 to 0.79)	0.095	0.59 (− 6.78 to 7.97)	0.874
Longitudinality	− 12.23 (− 16.25 to − 8.22)	< 0.001	− 12.00 (− 17.39 to − 6.61)	< 0.001
Coordination	− 0.18 (− 5.79 to 5.44)	0.951	5.59 (− 1.49 to 12.68)	0.123
Comprehensiveness (services available)	− 8.36 (− 14.15 to − 2.57)	0.005	− 7.17 (− 14.07 to − 0.27)	0.043
Comprehensiveness (services provided)	0.65 (− 5.36 to 6.65)	0.833	2.87 (− 4.69 to 10.42)	0.458
Community orientation	− 17.35 (− 22.10 to − 12.60)	< 0.001	− 17.08 (− 22.75 to − 11.42)	< 0.001

*JPCAT* Japanese version of Primary Care Assessment Tool

^a^All scores range from 0 to 100

^b^Adjusted for age, sex, years of education, and self-rated health status

For PWDs, 66% were male, 90% were aged < 70 years, and 70% had no college education. For non-disabled participants, 31% were male, 34% were aged < 70 years, and 55% had no college education.

Table 2 summarizes associations between disabilities and JPCAT scores.

After adjusting for age, sex, years of education, and self-rated health status, PWD scores were significantly lower than those of non-disabled participants for JPCAT total score, as well as Longitudinality and Community Orientation domain and Comprehensiveness (services available) subdomain scores. The largest mean difference between non-disabled participants and PWDs was found in the Community Orientation domain (adjusted mean difference: -17.08; 95% confidence interval (CI), − 22.75 to − 11.42), and the smallest mean difference was observed in the First Contact domain (adjusted mean difference: 0.59; 95% CI: − 6.78 to 7.97).

### Qualitative analysis

Although we initially adopted a deductive approach for extracting themes in using JPCAT domains (First Contact, Longitudinality, Coordination, Comprehensiveness, and Community Orientation), we added the new theme, “Accountability,” because there were many comments by participants that applied to this theme. Within these six themes, quotes from free-response answers were categorized as being either disability-specific or general, the latter of which was shared between PWDs and non-disabled participants.

### First Contact

Mean scores for the First Contact (Accessibility) domain did not significantly differ between PWDs and non-disabled participants (adjusted mean difference, 0.59; 95% CI: − 6.78 to 7.97). We did not extract a general theme for this domain in the qualitative analysis, but disability-specific themes were extracted, as reflected in the comments below.

*I have been undergoing follow-ups with this doctor for a long time. I need to drive the car for around 30 minutes to visit the hospital; however, in emergencies, such a long drive would be impossible. I believe that even hospitals dealing with emergencies have a difficult time accepting patients in wheelchairs.*

Another female PWD wrote:

*After I underwent surgery at A-City Hospital, I was transferred to the A-City Center of Regional Rehabilitation. I was followed up there on an outpatient basis after discharge. However, this rehabilitation center was closed by the wishes of the A-City administration in 2015. Although I can visit the Urology Department of a local clinic (I can also visit it for other illnesses, like colds), I am having a very difficult time finding other hospitals or clinics.*

Both PWDs expressed concerns about the inaccessible environment and lack of nearby service availability.

### Longitudinality

The mean score for the Longitudinality domain was significantly lower in PWDs compared to that in non-disabled participants (adjusted mean difference, − 12.00; 95% CI: − 17.39 to − 6.61). The qualitative analysis revealed a lack of continuity as a general theme in the Longitudinality domain, as reflected in the quote “at the university hospital, every three to five years, doctors are shuffled.” Another PWD who experienced the Great East Japan Earthquake in 2011 and evacuated from home noted:

*I have been transported to the hospital in B Prefecture five times due to extreme stress after the nuclear accident (which occurred on March 11, 2011). Two and half years later (October 2013), I was evacuated to K city (in a different prefecture).*

This PWD was one of many earthquake evacuees from B prefecture whose continuity of care was halted due to the nuclear accident in 2011. Several PWDs noted instances of disability-specific continuity. For example, a male PWD who had injured his spinal cord at the age of 30 stated that “it has been 24 years since the house entrance had a slope for my wheelchair, and after my 50 s, I now feel the slope is steep.” Many other PWDs felt a lag in continuity due to the Japanese insurance system for PWDs. Specifically, at the age of 65, a PWD faces a switch in insurance from HWSPWD to LTCI.

*When people with disabilities turn 65 years of age, they will generally start using long-term care insurance benefits. The government recommends such a system; however, I do not want to use it because I will have to pay more money to use it, and the hours of services I receive will be reduced.*

A female PWD was puzzled to receive a letter for seniors and pointed out that the change of insurance based on age made her aware of the shift in social status, i.e., shifting from a PWD to an elderly individual.

*I turned 65 last year. [One day] the application form of the long-term care insurance program arrived, and the envelope indicated the sender was the Senior Citizens’ Welfare Division. I was initially confused ... and wondered whether they sent it to the wrong address. I was unable to accept that it was indeed for me. It did not feel right that, at the age of 65 years, I am being considered a healthy, albeit senior, citizen without disabilities. I could not believe this sudden change in status [because] I have been a person with a disability since birth, and it should not be possible for the disability status to go away. I have pride in being a person with a disability, and in fact, it is my life.*

The shift in insurance coverage from one for PWDs to that for the elderly was accompanied by a transformation in identity. This PWD refused to blend into the new category of “elderly,” and was determined to live her life as who she is. Quotes in this section reflected a disruption of continuity prompted by a change in insurance.

### Coordination

Mean scores for the Coordination domain did not significantly differ between PWDs and non-disabled participants (adjusted mean difference, 5.59; 95% CI: − 1.49 to 12.68). In the qualitative analysis, one PWD noted:

*As we get older, we often go to different departments [in other clinics]. Under such circumstances, I would be grateful if these clinics are in coordination. It is best for one primary doctor to comprehensively manage my medical condition, but this appears to be difficult to achieve.*

As one ages, coordination of care becomes a concern, regardless of whether the person has a disability or not. Several other participants made disability-specific coordination remarks, such as “although the government promotes having a primary care physician, there are very few doctors who know about care for PWDs” or “it is very inconvenient that we don’t have a doctor or clinic which specializes in care for PWDs.” Another PWD noted:

*I believe there is good coordination between my primary physician and the specialist at the hospital. However, I need to manage my condition because the specialist knows only a particular field.*

These participants noted that, in addition to general care, disability-specific care is needed, and good coordination is required for both types of care.

### Comprehensiveness

Results of the quantitative analysis for the Comprehensiveness domain were mixed. While the Comprehensiveness (services available) subdomain score was significantly lower in PWDs compared to that in non-disabled participants (adjusted mean difference, − 7.17; 95% CI: − 14.07 to − 0.27), the Comprehensiveness (services provided) subdomain score did not significantly differ between the two (adjusted mean difference, 2.87; 95% CI: − 4.69 to 10.42).

In the qualitative analysis, one female PWD noted a general Comprehensiveness (services available) theme concerning end-of-life issues related to dementia, as follows:

*All members of my family have died. Thus, I feel anxiety about how my affairs and belongings will be handled in the event of my death or if I develop a condition like dementia.*

There were many mixed aging-related comments (both general and disability-specific) for the Comprehensiveness (services available) subdomain. A general theme included “whether or not you have a disability, as you grow older, you will become disabled.” On the other hand, other participants felt that “aging for PWDs is different from how the non-disabled get old” or “I think that the aging of PWDs will start two or three times faster than the aging of healthy people.” In line with the results from the quantitative analysis, in which PWDs had a significantly lower score for the Comprehensiveness (services available) subdomain compared to non-disabled participants, a male PWD with cerebral palsy “was surprised because he couldn’t predict that he wouldn’t be able to move physically around after becoming 65 years old.” He further noted:

*Although I often had heard from my friends with CP, there were many things that I couldn’t understand until I actually aged. The nearby internal medicine doctor does not seem to know about cerebral palsy. It is difficult to find a doctor who specializes in secondary disabilities derived from aging…So I go along with my body while deceiving it.*

Comprehensiveness (service available) seemed to be hampered by the lack of health care professionals with disability-specific care knowledge. Some PWDs may not have sought a doctor regarding a secondary disability because “I only talk about the disease with him (his doctor). He has never talked about social topics and it never occurred to me that I would mention them (social topics) to him.”

Comprehensiveness (services provided) subdomain scores did not significantly differ between PWDs and non-disabled participants. Several PWDs used care services such as cooking and cleaning. In the past, family members of PWDs were expected to care for them; however, “unlike those days when a PWD had no choice but to be cared for by the family or to live in a facility for the disabled,” a male PWD noted that a paid care helper supported him so that he could avoid being dependent on his wife.

Under a disability-specific theme of the Comprehensiveness (services provided) subdomain, a male PWD who enjoyed wheelchair basketball and tennis for 42 years noted the following:

*At nearly 70 years of age, my physical strength began to decline, and the intervals between short-term hospitalizations were getting shorter, at 1–2 times a year. Every month, I visit my primary physician for regular follow-ups about my [post]-spinal injury condition.*

### Community orientation

The mean score for the Community Orientation domain was significantly lower in PWDs compared to that in non-disabled participants (adjusted mean difference, -17.08; 95% CI: − 22.75 to − 11.42).

We did not extract a general theme for this domain in the qualitative analysis. The disability-specific comment below was raised by a male PWD:

*I would like medical professionals to learn more about the characteristics of people with different types of disabilities and aspects of their independent life in several communities, in addition to specific issues related to aging populations, by taking nursing school or care worker courses.*

Compared to the other domains, the concept of Community Orientation appeared to be unfamiliar or of little interest to PWDs. For instance, one PWD briefly noted: “I don’t know about his (my doctor’s) community orientation activities, and am not interested.”

### Accountability

Although there is no Accountability domain in the JPCAT, many free-response answers referred to the theme of accountability. Therefore, we inductively added this theme, as it was evidently an important theme to PWDs. As with the some of the JPCAT domains, there were both general and disability-specific comments.

General themes often took the form of the phrase “regardless of whether they have disabilities or not…” One PWD participant noted:

*With increasing age, many people cannot do things that they used to be able to... I think it is normal that the amount of daily living support increases with age. I hope that increased interactions with and support for older adults with disabilities will lead to creating a better society.*

On the other hand, disability-specific accountability was also raised by several PWDs, as reflected in comments such as “more places and hospitals are needed for people whose lower limbs are paralyzed, like me.” Another PWD was deeply saddened by the current system in which “the pension alone will not pay for even minimum living expenses…our country is unfriendly to people with disabilities who aim to keep living with dignity.” This PWD hoped the system would be reformed to comply with Article 25 of the Constitution (discussed further below).

In addition to accountability for health care professionals and the government, some PWDs referred to “shared” accountability. As one PWD put it: “I think we need to study secondary disabilities from the late 30 s.” Another PWD who used LTCI to lead an active life advocated for creating assisted living facilities for PWDs. He was “determined to further the project … which was a long-pending task in which any person with a disability can lead a fulfilling life.” Another PWD, who was aware of differences between experiences of the aging disabled and aging problems of the non-disabled, wanted to fill the gaps in disability services that exist across municipalities. These PWDs called for accountability not only to society, but also to themselves as stakeholders in care.

## Discussion

PWDs tend to be at a disadvantage for receiving preventive medicine and medical check-ups due to environmental barriers and a lack of health care providers who are familiar with disabilities [6]. PX is an essential component for achieving quality health care. A systematic review of 55 studies [7] revealed that PX and clinical effectiveness dimensions, such as health outcomes, adherence to recommended medications, preventive care, and the use of health care resources, had positive associations with each other. PX has been a topic of interest globally and researched across different types of patients. However, PX of PWDs has never been assessed, even though this population is known as a disadvantaged group with respect to receiving health care. The present study is the first to assess PX of PWDs and compare them to PX of non-disabled participants.

Through both qualitative and quantitative analyses, the present study provides health care professionals, especially primary care physicians, a glimpse into the rarely studied PX of PWDs. The quantitative arm of the study found that while scores for the Longitudinality and Community Orientation domains and Comprehensiveness (services available) subdomain of the JPCAT in PWDs were significantly lower than those in NPDs, scores tended to be higher for the remaining domains/subdomain in PWDs (First Contact, Coordination, Comprehensiveness (services provided)).

### Aging into and with disability

Disability and aging research [17] differentiates between “aging into disability” and “aging with disability.” The former refers to a general population having a disability as a result of aging, and the latter to a group of people who acquired a disability earlier in life and grew older with the disability. The aging with disability literature proposes two hypotheses on how PWDs age: double jeopardy and age as leveler. These concepts were originally derived from research [18] on the aging of racial minorities. The double jeopardy hypothesis proposes that, as PWDs age, the negative impact of their minority status becomes stronger relative to when they were younger. The age as leveler hypothesis, in turn, refers to the aging process in which the disadvantaged status of PWDs in their younger years becomes less pronounced because “age acts as a leveler across social strata” [19]. Our quantitative analysis revealed the Comprehensiveness (services available) subdomain score to be the only aging-related item to be significantly lower in PWDs than in non-disabled participants. However, the qualitative analysis included free-response comments which were consistent with both hypotheses.

A qualitative study [20] of “successful aging” for PWDs reported four themes, one of which was promoting or maintaining physical health in the subdomains of “maintenance of current physical health” and “access to appropriate healthcare.” The latter subdomain encompasses components such as availability, accessibility, and appropriateness. In that study, focus-group interview participants mentioned their family physicians who knew less about disability conditions than their patients with disabilities [20]. As one participant noted: “[m]ost general practitioners don’t know where to send people and for what services there are. I have a general practitioner that I like and have gone to for years, but he says, ‘I am too busy to research polio. You research it and I’ll be glad to send you’.” Such sentiment was echoed by participants of the present study. For instance, several PWDs noted a lack of health care providers knowledgeable about disability conditions and considered it their own responsibility to manage highly individualized disability-specific issues such as secondary disability, which requires both gerontological and disability-specific knowledge. This may explain why the Comprehensiveness (services provided) subdomain score and Coordination domain score did not significantly differ between PWDs and non-disabled participants. Given their long-term experiences with disability, PWDs of the present study were more knowledgeable than their doctors and devised tactics to manage their own health, and thus may have been active participants in the health care context.

### General and disability-specific themes: similarities and differences

The quantitative analysis revealed that scores for the Longitudinality and Community Orientation domains and Comprehensiveness (services available) subdomain were significantly lower in PWDs compared to those in non-disabled participants. To our surprise, however, scores for other domains and subdomains, including First Contact, Coordination, and Comprehensiveness (services provided), were higher in PWDs than those in non-disabled participants, albeit not significantly so. Therefore, in analyzing qualitative data, we also analyzed comments from the perspective of similarities and differences between PWDs and non-disabled participants, i.e., in the form of general or disability-specific themes.

For “disability-specific” themes, comments frequently referred to the unique challenges PWDs face, such as the aforementioned lack of health care providers, especially doctors who are familiar with disabilities, and “the insurance transition at age 65.” This transition negatively impacts many PWDs, as they end up paying more for fewer services [21]. For example, HWSPWD consists of four support menus, and many PWDs under 65 use “mobility support” to achieve social participation or leisure activities. Yet, after age 65, those services are no longer available under LTCI. Another example illustrating the difference between HWSPWD and LTCI concerns the provision of equipment, such as a wheelchair. Under LTCI, PWDs receive bulkier “one-fits-all” wheelchairs, which are much heavier than those they were able to receive under HWSPWD.

These service gaps occur due to differences in the objectives of the particular type of insurance [21]. HWSPWD aims to facilitate social participation, inclusion, and independence of PWDs, while LTCI aims for elderly care and the prevention of inactive life disease (disuse syndrome). In 2008, HWSPWD was sued for unconstitutionality in 14 district courts on the grounds that the Benefit-received Principle adopted by HWSPWD violates the right to life and the right to pursue happiness of PWDs. One PWD’s push for welfare reform in order to comply with Japan’s constitution in the Accountability domain reflects the aforementioned social background of Japan.

According to the WHO [1], PWDs need both general health care (similar to the general population), including health promotion, preventive care, and access to primary care, as well as disability-specific care, for example, to treat pressure ulcers and urinary tract infections. Japan does not have a family doctor registration system. Therefore, each patient, with or without a disability, chooses a doctor who might not be specialized in family medicine or general practice. Lower scores in PWDs for some domains may be reflected in comments by some PWDs who noted that doctors may try to avoid discussions on non-biomedical topics.

Qualitative analysis of free-response answers revealed comments that described joys and concerns in life that were shared by PWDs and non-disabled participants across many domains, including concerns about having dementia, enjoying sports, or a sense of diminishing social strata between PWDs and non-disabled participants with age. For example, one participant noted:

*With increasing age, many people cannot do things that they used to be able to, regardless of whether they have disabilities or not. I think it is normal that the amount of daily living support increases with age.*

The above comment from the Accountability domain represents the aforementioned “age as leveler” theme [19]. While PWDs face unique challenges due to their disabilities and social handicaps, they also share many common threads with non-disabled participants which tend to be disregarded or are unacknowledged in disability research. Accordingly, we made efforts in the present study to touch on common themes between PWDs and non-disabled participants as well.

Spinal Injuries Japan provides a comprehensive insurance switch manual to assist PWDs [22]. For instance, the manual provides information regarding the benefits of using LTCI service providers over HWSPWD service providers if the disability is not severe. Therefore, our recommendation is for insurance plan changes to be made based on disability status rather than age. We also encourage PWDs to become more familiar with currently available insurance systems, and to participate in information circles to stay up-to-date with the latest disability-related news.

### Strengths, limitations, and future directions

A major strength of the present study was the use of a quantitative/qualitative mixed-methods approach to examine the relatively unstudied topic of PX of PWDs in Japan. According to Chow et al. [23], “quantitative data may assist in providing the big picture, but it is the personal story, accompanied by thoughts and feelings, that brings depth and texture to the research study.” Also, we used a reliable outcome measure (JPCAT scores) for the quantitative part of the study, as well as free-response answers for the qualitative part, which provided context for the quantitative results. In addition to the mixed-methods design, the results were double-checked by a team of researchers from various disciplines and methodology expertise to increase the internal validity of the results.

There are also some limitations worth noting. First, the qualitative part of the study relied on optional free-response answers by 54 of the 169 PWDs. Those who did not have strong views on the issue were likely to have left the space blank, and only those with strong views, in particular, negative ones, may have provided comments. Second, PWDs of the present study were members of disability organizations such as those for spinal cord injuries or the Center for Independent Living (CIL). One study [20] pointed out that having social support and connections with not only the general but also the disability community is key for successful aging. In this regard, our participants may have been immersed in a disability network, in which self-care techniques, peer-support, and information about insurance updates were plentiful. This unique setting may have influenced the results. Third, the low response rate of the survey (36% for PWDs) should be noted. Finally, the data used in this study were collected prior to the COVID-19 pandemic. Hence, the situation may be quite different now and in the foreseeable future.

Future research can build on the present findings in many ways. For instance, many PWDs noted they had no physicians who were familiar with disability-specific care, especially for PWDs with a secondary disability. Health care needs for secondary disability uniquely intersect at disability-specific conditions and the general aging trajectory, and needs to gather further attention among general practitioners. Research on disability [20] suggests that demographic conditions such as age, sex, and/or economic status contribute to differences in PWD experiences, including care. WHO reports that women with disabilities and PWDs in high-income countries seek care more often than men with disabilities and PWDs in low-income countries [1]. However, as the aim of the present study was to compare PWDs and non-disabled participants, we did not touch on within-group differences among PWDs. Further studies will be needed to examine differences that exist within the disability group based on demographic traits. Finally, many participants expressed concerns about the insurance switch that occurs at age 65. This may also be a topic for further research, e.g., if and how the insurance switch affects social participation, relationships, and/or health care services of PWDs.

## Conclusion

The present study investigated PX among Japanese PWDs, and found that JPCAT scores were significantly lower in PWDs than in non-disabled participants for the Longitudinality and Community Orientation domain and Comprehensiveness (services available) subdomain. Qualitative analysis revealed common themes with non-disabled participants, as well as unique challenges faced by PWDs, such as the lack of health care providers familiar with disabilities and the insurance transition at age 65, a unique feature of the Japanese health care system.

## Data Availability

The datasets generated and analyzed during the present study are not publicly available due to confidentiality but are available from the corresponding author on reasonable request.

## Abbreviations

HWSPWD: Health and Welfare Services for Persons with Disabilities
JPCAT: Japan Patient Experiences of Primary Care
LTCI: Long-term Care Insurance
PROGRESS: Primary Care Organizations Reciprocal Evaluation Survey Study
PWDs: People with Disabilities
PX: Patient Experience
WHO: World Health Organization
